# Ultrasound-guided Supraclavicular Brachial Plexus Block for Therapeutic Management of Postoperative Compressive Brachial Plexus Neuropathy: A Case Report

**DOI:** 10.5811/cpcem.6600

**Published:** 2024-06-14

**Authors:** Daniela Usuga, Sofia Portuondo, David Farcy, Michael Shalaby

**Affiliations:** * Florida International University, Herbert Wertheim College of Medicine, Miami, Florida; † Mount Sinai Medical Center Miami Beach, Department of Emergency Medicine, Miami Beach, Florida; ‡ Florida International University, Herbert Wertheim College of Medicine, Department of Emergency Medicine and Critical Care, Miami, Florida

**Keywords:** Supraclavicular Brachial Block, ultrasound, compressive neuropathy, brachial plexus

## Abstract

**Introduction:**

Compressive neuropathy of the brachial plexus is a common issue following laparoscopic and robotic surgeries.

**Case Report:**

A 71-year-old male, post-lumbar spinal surgery, presented with excruciating right upper extremity pain and paresthesias. A supraclavicular brachial plexus (SBP) block with bupivacaine provided significant pain relief, lasting 36 hours. Subsequent physical therapy led to gradual pain and weakness improvement in compressive neuropathy.

**Discussion:**

The SBP block, facilitated by ultrasound guidance, is a safe procedure with few serious complications. It proves beneficial for managing postoperative compressive neuropathy, allowing patients to break pain cycles and participate in rehabilitation.

**Conclusion:**

The SBP block is an effective addition to the management of postoperative compressive neuropathy, given its ease, safety, and potency. Although regional anesthesia provides only temporary relief, patients can experience a break in debilitating pain cycles associated with compressive neuropathy.

Population Health Research Capsule What do we already know about this clinical entity?

*Brachial plexus compression is a common post-operative complication, exacerbated by prolonged procedures such as robotic surgeries in the Trendelenberg position.*

What makes this presentation of disease reportable?

*We report successful treatment of post-operative brachial plexus neuropathy with ultrasound-guided supraclavicular brachial plexus (SBP) block.*

What is the major learning point?

*Ultrasound-guided SBP block offers significant pain relief for post-operative compressive neuropathy, breaking pain cycles until outpatient management is feasible.*

How might this improve emergency medicine practice?

*Ultrasound-guided SBP block is a safe, effective option to break pain cycles, enabling therapy and enhancing post-operative compressive neuropathy management.*

## INTRODUCTION

The brachial plexus arises from the spinal nerve roots of fifth cervical to first thoracic and supplies sensory and motor innervation to the upper limb and shoulder girdle. Compressive neuropathy, also known as entrapment neuropathy, occurs from the compression of a nerve and can result in temporary or permanent weakness and pain, which can be debilitating.^1^ The brachial plexus is located in the posterior triangle in the neck and passes proximally between the scalene muscle and distally between the first rib and clavicle.^2^ With increased utilization of laparoscopic and robotic surgery, there has been an increased incidence of brachial plexus neuropathy,^3^ especially given the prolonged time that the patient spends in the Trendelenberg position.^2^ ^–^ ^4^

Previously published case reports and case series have reported brachial plexus neuropathy following prolonged spinal surgery.^5^ ^–^ [Bibr r6] ^7^ Spinal surgeries in which the patient is in the prone position with their arms abducted at an angle greater than 90° have been demonstrated to have significantly increased incidence of postoperative brachial plexus injury.^6^ Such cases have also been replicated in animal studies, in which interrupted blood flow to or prolonged stretching of the brachial plexus results in intraneural capillary rupture and hematoma formation.^4^

Parsonage-Turner syndrome, also known as idiopathic brachial plexopathy or neuralgic amyotrophy, is a rare condition characterized by a diverse range of symptoms.^8^ It typically manifests with sudden onset shoulder pain on one side, followed by progressive neurological issues such as motor weakness, dysesthesias, and numbness. While the exact cause of the syndrome is not well understood, it has been observed in various clinical scenarios, including postoperative compressive, postinfectious, post-traumatic, and post-vaccination settings.^8^

## CASE REPORT

Our patient was a 71-year-old man who had a fourth lumbar to fifth lumbar posterior lumbar interbody fusion with third lumber to fifth lumbar posterolateral decompression and fusion one month before presentation. He presented to our emergency department (ED) with a chief complaint of painful paresthesias of the entire right upper extremity. After remaining on his right lateral decubitus for an extended period intraoperatively, the patient subsequently developed painful paresthesias in the right shoulder and right arm. He had seen a neurologist in the clinic, who prescribed gabapentin 300 milligrams (mg) three times daily and methocarbamol 750 mg three times daily for compressive neuropathy. However, the patient had achieved minimal analgesia with this regimen, and he rated his pain as a 10/10 upon presentation to the ED. The patient’s physical exam showed intact strength and reflexes in upper extremities bilaterally. However, he had decreased sensation to sharp touch over the entire upper extremity compared to the left.

The patient consented to a supraclavicular brachial plexus (SBP) block, which was performed with 15 milliliters (mL) bupivacaine 0.5% with epinephrine. He tolerated the procedure well and was pain-free when discharged. At follow-up via phone call one week later he stated that he’d had 36 hours of pain relief from the brachial plexus block. Since the procedure, the patient has been in physical therapy and his pain from his compressive neuropathy has been slowly improving.

## DISCUSSION

An ultrasound (US)-guided SBP block involves instilling anesthetic within the nerve sheath to anesthetize the upper, middle, and lower trunks of the brachial plexus. The SBP can typically be visualized by placing a short linear US probe within the supraclavicular fossa, the space immediately posterior to the middle to medial clavicle (Image). In this view, the subclavian artery is viewed medial to the SBP, the first rib caudal, and the pleura deep to the first rib (Figure).

**Image.**
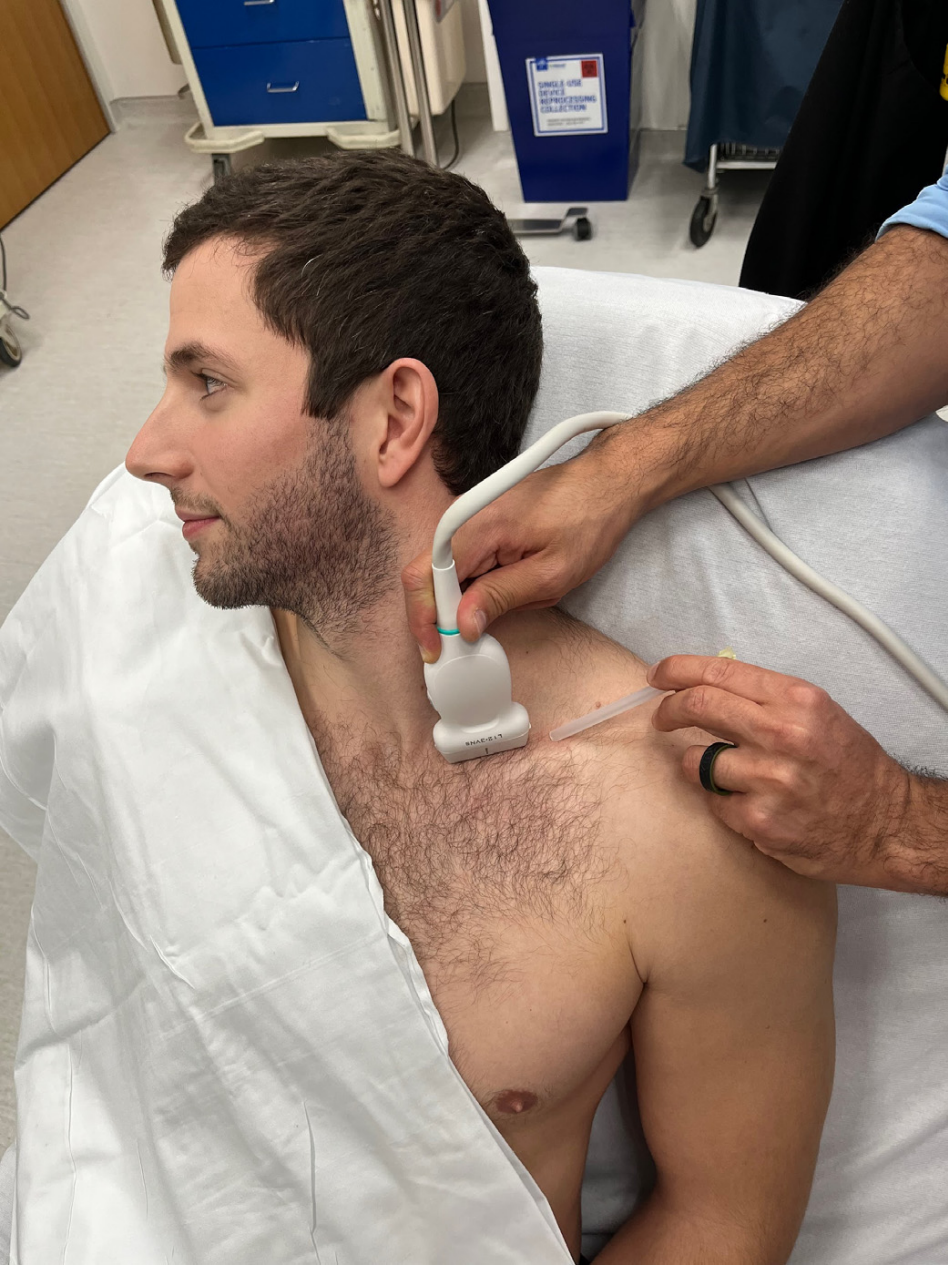
Ultrasound probe placement in the supraclavicular fossa to identify and anesthetize the supraclavicular brachial plexus. The needle is shown before introduction from a lateral to medial approach (shown here on a model patient).

**Figure.**
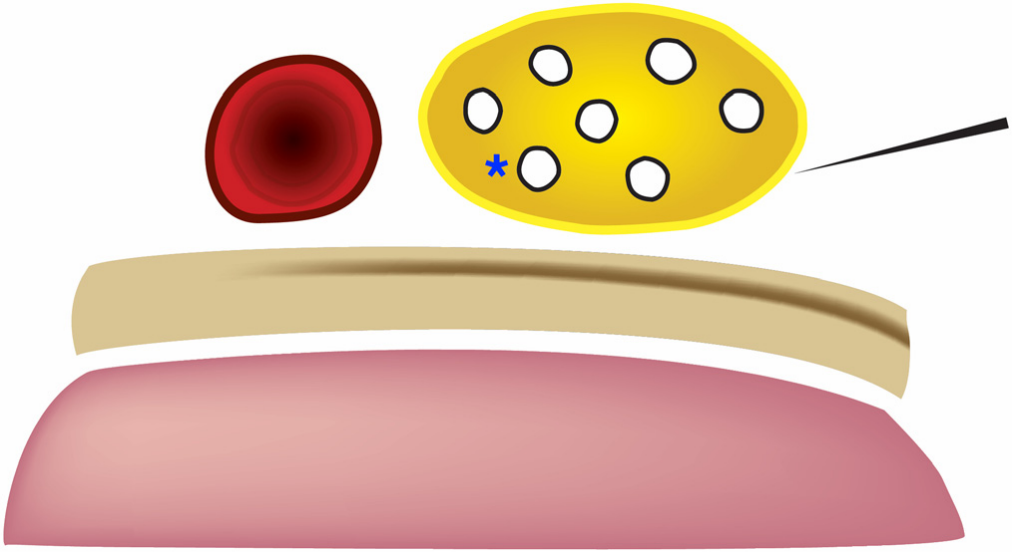
Graphic demonstrating the supraclavicular brachial plexus (yellow), subclavian artery medial (red), first rib deep (beige), and pleura (pink). The needle shaft makes a shallow angle towards the brachial plexus. The blue asterisk represents the “corner pocket.”

With an in-plane needle technique, the physician uses a shallow needle approach from lateral to medial to guide the needle toward the SBP. Once the needle is clearly visualized within the SBP sheath, the physician slowly injects aliquots of 5 mL of anesthetic at a time, making sure to aspirate prior to any injection. Furthermore, injecting at the “corner pocket” (Figure) closest to the subclavian artery helps to guarantee complete anesthesia of the inferior trunk of the brachial plexus.^9^ The SBP is very superficial, typically 1–2 centimeters deep in the skin; thus, a 22-gauge, non-spinal needle can usually be used for this procedure. The Miller weight-based local anesthetic dosing should be used to calculate the ideal anesthetic dosage based on ideal body weight.^10^ However, 15–20 mL of local anesthetic is usually adequate for anesthesia of the upper limb. The first rib serves as a backstop, should the physician accidentally overshoot the SBP, to avoid causing a pneumothorax.^10^ Besides pneumothorax, other complications of the SBP include axonal damage, hemidiaphragmatic paralysis, and subclavian artery puncture.^10^

The use of US to perform a SBP block, as opposed to the landmark-based technique, significantly reduces the likelihood of pneumothorax and neuronal injury, as reported in previous studies.^11^ ^,^ ^12^ Additionally, should the performing physician maintain proper needle control throughout the procedure, the pleura and subclavian artery remain comfortably outside the needle’s trajectory to the SBP.^9^ By anesthetizing all trunks of the brachial plexus, the SBP block provides reliable anesthesia of most of the upper extremity, including the shoulder, but sparing the upper medial arm (which is innervated by the second thoracic spinal nerve).^13^ ^,^ ^14^ ^,^ ^15^

The brachial plexus has increased vulnerability to injury due to its superficial location in the neck and to patient positioning during certain prolonged surgical procedures. While our patient had not yet received a formal diagnosis of Parsonage-Turner Syndrome by his neurologist, his symptoms were consistent with the disease. Although chronic pain resulting from compressive neuropathy often fluctuates in intensity, our patient experienced excruciating pain, which caused him to present to the ED that day. While regional anesthesia does not provide a permanent solution, it can afford a substantial amount of relief in the short term and break pain cycles.

Subsequently, our patient was able to resume his physical therapy without revisiting the ED due to pain. For patients grappling with similar debilitating pain caused by compressive neuropathy, regional anesthesia can be a safe and effective option. Furthermore, per the American Society of Regional Anesthesia, post-surgical compressive neuropathy is not a contraindication to treatment with regional anesthesia.^16^ The SBP block is feasible for emergency physicians to perform at the bedside, and the use of US significantly reduces complications.^6^ ^,^ ^16^ ^–^ ^19^

## CONCLUSION

Considering the ease, safety, and potency of the SBP block, emergency physicians should include the SBP block in the multimodal approach to the management of postoperative compressive neuropathy. While pain from compressive neuropathy is chronic, it also waxes and wanes and at times can become debilitating. Thus, regional anesthesia in general can provide significant relief for patients experiencing similar pain.

## Supplementary Information

Video. Performance of an ultrasound-guided supraclavicular brachial plexus block.
*SA*, subclavian artery; *BP*, brachial plexus; *NS*, needle shaft.
